# Unraveling the Cardiac Matrix: From Diabetes to Heart Failure, Exploring Pathways and Potential Medications

**DOI:** 10.3390/biomedicines12061314

**Published:** 2024-06-13

**Authors:** Bogdan-Sorin Tudurachi, Larisa Anghel, Andreea Tudurachi, Radu Andy Sascău, Răzvan-Liviu Zanfirescu, Cristian Stătescu

**Affiliations:** 1 Internal Medicine Department, “Grigore T. Popa” University of Medicine and Pharmacy, 700503 Iasi, Romania; bogdan-sorin.tudurachi@d.umfiasi.ro (B.-S.T.); radu.sascau@umfiasi.ro (R.A.S.); cristian.statescu@umfiasi.ro (C.S.); 2 Cardiology Department, Cardiovascular Diseases Institute “Prof. Dr. George I. M. Georgescu”, 700503 Iasi, Romania; leonteandreea32@gmail.com (A.T.); zanfirescu_razvan-liviu@d.umfiasi.ro (R.-L.Z.); 3 Physiology Department, “Grigore T. Popa” University of Medicine and Pharmacy, 700503 Iasi, Romania

**Keywords:** diabetes, myocardial infarction, heart failure, myocardial fibrosis, SGLT2 inhibitors, GLP-1 agonist, dipeptidyl peptidase-4 inhibitors

## Abstract

Myocardial infarction (MI) often leads to heart failure (HF) through acute or chronic maladaptive remodeling processes. This establishes coronary artery disease (CAD) and HF as significant contributors to cardiovascular illness and death. Therefore, treatment strategies for patients with CAD primarily focus on preventing MI and lessening the impact of HF after an MI event. Myocardial fibrosis, characterized by abnormal extracellular matrix (ECM) deposition, is central to cardiac remodeling. Understanding these processes is key to identifying new treatment targets. Recent studies highlight SGLT2 inhibitors (SGLT2i) and GLP-1 receptor agonists (GLP1-RAs) as favorable options in managing type 2 diabetes due to their low hypoglycemic risk and cardiovascular benefits. This review explores inflammation’s role in cardiac fibrosis and evaluates emerging anti-diabetic medications’ effectiveness, such as SGLT2i, GLP1-RAs, and dipeptidyl peptidase-4 inhibitors (DPP4i), in preventing fibrosis in patients with diabetes post-acute MI. Recent studies were analyzed to identify effective medications in reducing fibrosis risk in these patients. By addressing these areas, we can advance our understanding of the potential benefits of anti-diabetic medications in reducing cardiac fibrosis post-MI and improve patient outcomes in individuals with diabetes at risk of HF.

## 1. Introduction

The cardiac muscle, comprising cardiomyocytes, capillaries, and ECM rich in collagen fibers, constitutes the fundamental elements crucial for efficient contraction. Left ventricular (LV) remodeling, an adaptive response to mechanical, neurohormonal, and genetic stimuli, orchestrates alterations in ventricular dimension, shape, and function. While physiological cardiomyocyte proliferation in scenarios like pregnancy signifies a reversible process facilitated by augmented microcirculatory perfusion, pathological remodeling after cardiac events such as MI poses a substantial risk for HF [1]. HF, a complex syndrome stemming from structural and functional aberrations within the heart, manifests pathologically through interstitial fibrosis, chamber remodeling, and diminished ventricular compliance [2]. Diabetes mellitus (DM) emerges as an independent risk factor for HF, with diabetic cardiomyopathy representing a significant complication fueled by metabolic perturbations including glucose and lipotoxicity, insulin resistance, endothelial dysfunction, and microvascular impairment, alongside ischemic heart disease sequelae, involving multifaceted pathophysiological pathways [3]. Type 2 diabetes mellitus (T2DM) assumes a pivotal role in cardiovascular (CV) pathology, directly intertwining with diabetic cardiomyopathy and exacerbating CV risk factors, thus amplifying susceptibility to CAD. CAD-induced MI and ischemia/reperfusion injuries frequently instigate HF via acute or chronic maladaptive remodeling processes, establishing CAD and HF as principal contributors to CV morbidity and mortality. Consequently, therapeutic strategies for patients with CAD focus on averting MI occurrence and mitigating HF burden post-MI [4,5].

Traditionally, T2DM management commenced with insulin therapy; however, the evolution of oral anti-diabetic agents revolutionized glycemic control. Nonetheless, concerns regarding the CV safety profile of certain anti-diabetic drugs prompted extensive research aimed at elucidating their CV risk. Notably, studies unveiled two drug classes, SGLT2i and GLP1-RAs, which not only demonstrated CV safety but also exhibited remarkable reductions in major adverse CV events, including CV mortality, thereby positioning them as favorable options in T2DM management due to their low hypoglycemic risk and beneficial cardiovascular effects (CV benefits) [6]. Fibrosis, a chronic pathological process characterized by aberrant ECM deposition, serves as a hallmark feature across various cardiac pathologies, reflecting both reparative and maladaptive responses to injury. While initially pivotal for wound healing and tissue repair, persistent ECM accumulation engenders structural distortion and functional impairment within the heart. Myocardial fibrosis encompasses both interstitial and replacement forms, driven by diverse cellular and paracrine mechanisms involving inflammatory mediators, growth factors, and collagen-modulating enzymes such as matrix metalloproteinases (MMPs). Dysregulated collagen turnover precipitates myocardial fibrosis progression, further underscoring its pivotal role in cardiac remodeling [7].

**Figure 1:**
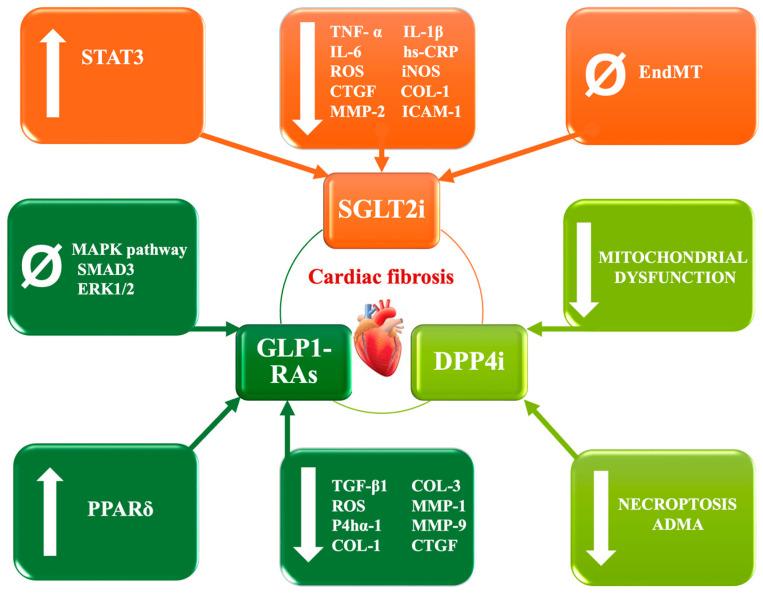
Pathways and potential medications related to cardiac fibrosis. ↑ = increase; ↓ = decrease; ∅ = inhibit; STAT3 = signal transducer and activator of transcription 3; TNF-α = tumor necrosis factor-α; IL-1β = interleukin-1β; IL-6 = interleukin-6; hs-CRP = high sensitivity c-reactive protein; COL-1 = type 1 collagen; COL-3 = type 3 collagen; MMP-1 = matrix metalloproteinase 1; MMP-2 = matrix metalloproteinase 2; MMP-9 = matrix metalloproteinase 9; PPARα = peroxisome proliferator-activated receptor alpha; DPP4i = dipeptidyl peptidase-4 inhibitor; SGLT2i = sodium-glucose cotransporter 2 inhibitors; GLP-1RAs = glucagon-like peptide-1 receptor agonists; CTGF = connective tissue growth factor; iNOS = inducible form of nitric oxide synthase; ROS = reactive oxygen species; ICAM-1 = intercellular adhesion molecule 1; MAPK = mitogen-activated protein kinase; ERK1/2 = extracellular signal-regulated kinase 1/2; EndMT = endothelial-to-mesenchymal transition; TGF- β1 = transforming growth factor-β; P4hα-1 = prolyl 4-hydroxylase subunit alpha 1; and ADMA = asymmetric dimethylarginine.

Understanding key pathophysiological processes holds paramount importance for identifying novel therapeutic targets and leveraging diagnostic and prognostic modalities, including biomarkers and imaging techniques, to tailor medical interventions, thereby enhancing patient outcomes and clinical management strategies (Figure 1).

## 2. Materials and Methods

Myocardial fibrosis, often a leading cause of sudden cardiac death, occurs due to an accumulation of ECM, sometimes called interstitial myocardial enlargement. Myocardial ischemia/reperfusion (MI/R) injury damages cardiomyocytes, triggering fibroblast activation and fibrosis formation. In MI, “interstitial fibrosis” emerges as collagen-based scars that replace necrotic cardiac cells from ischemia. Another aspect, “perivascular fibrosis”, involves the thickening of microvascular adventitia. While dilated cardiomyopathy (DCM) does not typically result in immediate cardiomyocyte loss, abnormal metabolism can lead to their atypical demise, causing “replacement fibrosis” that worsens DCM. Although there is an established connection between cardiac fibrosis and acute myocardial infarction (AMI) as well as diabetes, the current research on the correlation between emerging anti-diabetic medications and this phenomenon is insufficient. Therefore, the present study aims to examine the inflammation role in cardiac fibrosis and the efficacy of drugs like SGLT2i, GLP-1RAs, or DPP4i in preventing fibrosis in patients with diabetes following acute MI [2,8,9]. We conducted a narrative review incorporating contemporary studies from the last five years, published in MEDLINE, PubMed, and EMBASE databases, with the goal to identify effective medications in reducing fibrosis risk in these patients.

## 3. Collagen Synthesis

### 3.1. C-Terminal Propeptide of Procollagen Type I

The C-Terminal Propeptide of Procollagen Type I (PICP) is released into the bloodstream when procollagen type I carboxy-terminal proteinase cleaves it from the protein to produce fibril-forming collagen type I. In individuals without liver cirrhosis, PICP serves as a marker of collagen type I production and is metabolized by the liver [10]. Elevated levels of PICP have been observed in the blood of patients with heart failure and hypertension, correlating with myocardial thickening and impaired heart relaxation in those with arterial hypertension [11]. Prior to receiving medication therapy, hypertensive individuals have notably higher levels of PICP in their bloodstreams [12]. Currently, there is no evidence indicating a relationship between PICP levels and the progression towards heart failure in individuals experiencing acute ischemic events who also have diabetes.

### 3.2. Procollagen Type I N-Terminal Propeptide

The Procollagen type I N-Terminal Propeptide (PINP), indicative of collagen type I production, is generated through the conversion of collagen type I. Unlike PICP, PINP is characterized by its delayed release. In a case–control study, it was revealed that patients with HF had significantly higher baseline serum PINP levels compared to controls. Furthermore, another study observed a notable increase in plasma PINP levels in the MI group compared to the normal control group in rats with ischemic cardiomyopathy [13,14].

### 3.3. Procollagen Type III Amino-Terminal Propeptide

Procollagen Type III Amino-Terminal Propeptide (PIIINP) is released into the bloodstream as a result of increased collagen type III production during cardiac fibrosis, and it plays a crucial role in myocardial elasticity. A study on DCM revealed that patients with idiopathic or ischemic DCM exhibited higher baseline blood PIIINP levels compared to healthy individuals. Additionally, in rats with a MI model, the reduction in fibrosis attributed to tanshinone IIA was associated with a decrease in serum PIIINP levels [7,15]. Osokina et al. demonstrated that the risk of cardiac fibrosis increases when PIIINP levels reach or exceed 381.4 ng/mL on the 12th day after ST-segment elevation myocardial infarction (STEMI) with maintained left ventricular ejection fraction (LVEF). Monitoring PIIINP levels during hospitalization may aid in identifying individuals at high risk of cardiac fibrosis one year after STEMI with normal LVEF [16].

## 4. Collagen Degradation

### 4.1. C-Terminal Telopeptide of Collagen Type I

As a marker of collagen turnover and a potential indicator for the early detection of adverse remodeling, C-terminal telopeptide, also referred to as beta-CTx, is a fragment of Type I collagen released into circulation following its breakdown. According to Zelniker et al., in individuals with non-ST-elevation acute coronary syndrome (NSTEMI), beta-CTx levels were associated with both HF and CV mortality [17]. Manhenke et al. suggested that, in patients experiencing AMI, plasma beta-CTx served as an independent predictor of CV death, with levels rising in those who died from any cause [18].

### 4.2. Matrix Metalloproteinases

The extracellular matrix is viewed as a dynamic and vital framework essential for cardiac healing processes. MMPs are zinc ion-dependent proteases responsible for collagen and proteoglycan breakdown, playing a critical role in atherosclerosis progression. MMPs also play a pivotal role in post-AMI cardiac remodeling and the development of associated complications, with the myocardium expressing numerous MMPs [19,20]. Research suggests that matrix metalloproteinase 28 (MMP-28) is associated with CV events and mortality both during hospitalization and within 30 days after discharge, serving as a potential predictor of short-term prognosis for individuals with MI [21]. Recent studies have shown that young patients experiencing STEMI exhibit significantly elevated levels of matrix metalloproteinase 3 (MMP-3), matrix metalloproteinase 9 (MMP-9), and tissue inhibitors of metalloproteinase (TIMP) compared to healthy individuals. Those experiencing Major Adverse Cardiovascular Events (MACEs), particularly HF hospitalizations, demonstrate markedly increased levels of MMP-9, suggesting its involvement in cardiac remodeling and its impact on LV function [22]. Furthermore, elevated MMP-9 levels have been associated with a notable increase in CV mortality over a two-year period, with patients with advanced HF being more prevalent in the high MMP-9 group during the monitoring period [23].

Watson et al. propose that the minor allele of genetic variation rs3918242 in the promoter region of the MMP-9 gene may be linked to hypertension and/or MI. Patients with diabetes patients carrying the minor T allele of rs3918242 showed a higher likelihood of experiencing MI, reduced ejection fraction (EF), and increased progression of LV systolic dysfunction over a 3.5-year follow-up period, indicating an elevated risk for developing HF [24].

### 4.3. Tissue Inhibitors of Metalloproteinases

TIMPs constitute a subgroup of small glycoproteins synthesized and released by fibroblasts, epithelial cells, and endothelial cells, prevalent in various tissues. They play a role in initiating the conversion of fibroblasts into myofibroblasts at sites of tissue damage and regulate the enzymatic activity of MMPs by inhibiting the activation of latent MMPs. Thus, maintaining an appropriate balance between MMPs and TIMP production is critical for the reconstruction of the extracellular matrix in cardiac tissues. Four types of TIMPs have been identified to be associated with myocardial fibrosis [7].

A retrospective cohort study has indicated that blood levels of tissue inhibitors of metalloproteinase 3 (TIMP-3), CA125, and NT-proBNP can predict ventricular remodeling in patients with HF following AMI. These three markers demonstrated excellent specificity and moderate sensitivity. Early detection of serum TIMP-3, CA125, and NT-proBNP levels may enhance the accuracy of predicting cardiac remodeling and facilitate early detection and management of HF post-AMI [25].

Clinical investigations have shown that MMP and TIMP levels during the acute phase may predict the development of HF or CV events within a 30-day follow-up period in patients with AMI who did not receive reperfusion treatment. Specifically, plasma levels of tissue inhibitors of metalloproteinase 1 (TIMP1) and the matrix metalloproteinase 2 (MMP2)/TIMP1 ratio can indicate the likelihood of HF and CV events. The matrix metalloproteinase 8 (MMP8)/TIMP1 ratio has shown predictive utility for the risk of CV-related events [26].

## 5. Collagen Metabolism

### 5.1. Transforming Growth Factor-Β and SMADS

Transforming growth factor-β (TGF-β) is a crucial cytokine involved in promoting fibrosis within the heart muscle. It regulates the fibroblast growth, differentiation, migration, and synthesis of the ECM. The mammalian TGF-β subfamily comprises three isoforms (TGF-β1, 2, and 3) that bind to identical receptors and target similar cellular substrates. However, they exhibit distinct regulatory patterns and different affinities for their receptors and co-receptors, suggesting unique functions in fibrotic disease development [2].

TGF-β induces fibrosis in cardiac fibroblasts by activating the Smad3, PI3K/Akt, and mitogen-activated protein kinase (MAPK) signaling pathways as a cytokine upstream of the oxidized low-density-lipoprotein receptor (LOX) [7]. Following MI, there is a significant increase in TGF-β1 expression, which may interact with the renin–angiotensin system to promote heart remodeling. TGF-β1 stimulates the activation of fetal genes in myocardial cells and induces fibroblasts in the heart to produce ECM proteins.

Research by Zhang et al. investigated the effects of TGF-β1 therapy on myocardial cell death in rats with MI. AMI was induced in rats by occluding the left anterior descending coronary artery. Both the model and control groups were assessed for mRNA and protein expression levels of TGF-β1 in myocardial cells. AMI rats exhibited significantly higher levels of TGF-β1 mRNA and protein expression compared to the control group. The study suggested that the TGF-β1/MAPK signaling pathway was activated in myocardial cells during AMI, and administration of TGF-β1 was shown to attenuate myocardial cell death in AMI rats [27].

TGF-β transmits signals through intracellular effector proteins called Smads, as well as through Smad-independent pathways. In vivo research indicates that Smad3 signaling plays a crucial role in activating reparative fibroblasts after MI, leading to ECM protein synthesis and integrin transcription, which contribute to scar formation. Collagen levels decreased in the infarcted hearts of Smad3-deficient animals 7 days post-reperfusion, with reduced collagen deposition in the non-infarcted, remodeling myocardium. Smad3 signaling in myofibroblasts within the infarcted area inhibits cell proliferation, resulting in organized clusters of activated myofibroblasts and maintaining structural integrity through integrin and reactive oxygen species (ROS)-mediated pathways [2,7,28]. Disruption of the Smad gene appears to attenuate the remodeling process.

### 5.2. Corin

Corin, a transmembrane serine protease of type II, is predominantly found in atrial cardiomyocytes. It is a complex protein comprising a transmembrane domain near the N terminus, two frizzled-like domains, eight low-density lipoprotein (LDL) receptor repeats, a scavenger receptor-like domain, and a trypsin-like protease domain at the C terminus. Corin plays a crucial role in converting pro-atrial natriuretic peptide (pro-ANP) into its active form, ANP. Numerous studies have indicated that circulating corin levels have predictive value regarding various CV outcomes in individuals with AMI, chronic HF, and acute stroke [29,30,31,32]. However, preliminary findings from a small study suggest that serum levels of PCSK6 and corin are not independent predictors of CV outcomes in patients undergoing coronary angiography. Nonetheless, elevated blood PCSK6 levels in patients with non-chronic kidney disease (CKD) may indicate a higher risk of CV events [32]. To elucidate the critical genes and pathways involved in abnormal myocardial remodeling following prolonged ischemia, a study utilized a rhesus monkey model of MI. It revealed that cardiac remodeling may be aggravated by an imbalance in the expression of corin and natriuretic peptide A/natriuretic peptide B [33].

## 6. Inflammatory Factors

### 6.1. Galectin-3

The lectin family includes Galectin-3 (Gal-3), which has been associated with myocardial fibrosis, acute CV events, autoimmune diseases, and various inflammatory conditions. Animal models and a limited number of human samples have demonstrated its significant role in tissue healing following AMI. Gal-3 is primarily produced by activated macrophages and injured cardiomyocytes, highlighting its critical involvement in myocardial fibrosis. Elevated Gal-3 expression has a strong correlation with the onset and progression of CV disorders, making it closely linked to the adverse remodeling of the heart and MACEs [34]. MACE remains the leading cause of morbidity and mortality in patients with AMI, and several studies suggest that Gal-3 plays a significant role in predicting adverse events post-AMI [34,35,36,37,38,39]. According to research by Grandin E. and colleagues, patients with elevated levels of both BNP and Gal-3 have the highest likelihood of developing HF, suggesting that Gal-3 may provide additional value in assessing HF risk after acute coronary syndrome (ACS) [40].

Increased levels of fibrosis and indicators of ECM remodeling, such as collagen, fibronectin, TGF-β, fibroblast proliferation, alpha smooth muscle actin (αSMA), and MMPs, have been associated with peak levels of Gal-3 in the ischemic region [41,42]. In addition to promoting ECM remodeling, Gal-3 may facilitate neutrophil adhesion and recruitment to the injured heart. While recent research suggests that neutrophils contribute to healing and the temporal progression of MI, their involvement is typically limited to the first week post-AMI. Neutrophils can alter their phenotype during the healing process, transitioning from a pro-inflammatory to an anti-inflammatory profile and contributing to scar formation [43,44,45].

In a rat model of MI, the inhibition of Gal-3 led to decreased collagen synthesis and reduced myocardial fibrosis [46]. Additionally, through its dual roles as a chemotactic factor for macrophage recruitment and a regulator of their polarization towards the reparative M2 phenotype, Gal-3 plays a multifaceted role in post-infarction wound healing. Gal-3′s dual function is crucial for coordinating the immune response and tissue repair mechanisms after myocardial infarction, ultimately aiding in the recovery of heart tissue [41,47]. Animals with Gal-3 knockdown and pharmacologic suppression of Galectin exhibited decreased fibrosis, apoptosis, inflammation, and infarct size [48,49].

Gal-3 is intricately linked to inflammation, fibrosis, remodeling, and dysfunction in CV pathophysiology. Its significant involvement in cardiac remodeling underscores the importance of understanding its role in cardiac function. Understanding how Gal-3 operates in the heart holds promise for therapeutic interventions aimed at reducing HF.

### 6.2. Tumor Necrosis Factor-α and Interleukins

Macrophages and monocytes can generate and release numerous pro-inflammatory mediators, including interleukin-1β (IL-1β), interleukin-4 (IL-4), interleukin-6 (IL-6), and tumor necrosis factor-α (TNF-α). These inflammatory factors collectively form a complex regulatory network that promotes fibrotic degenerative processes and the activation of myofibroblasts [50]. Kang et al. demonstrated elevated concentrations of TNF-α, IL-6, and IL-1β, as well as increased expression of collagen I and III mRNAs compared to normal controls, suggesting a correlation between fibrosis and inflammation [51]. Fang et al. conducted a cross-sectional study on hypertrophic cardiomyopathy (HCM) and observed a positive correlation between plasma levels of IL-4, IL-6, and interleukin-10 (IL-10) with both diffuse and localized myocardial fibrosis [52].

TGF-β plays a critical role in transitioning from inflammation to fibrosis by inhibiting inflammation and promoting ECM deposition. Studies have shown that mice lacking TGF-β in their heart muscle exhibit reduced cardiac fibrosis and increased survival rates [53,54,55]. The IL-6 trans-signaling cascade has been implicated in cardiac fibrosis induced by aldosterone. Conversely, blocking IL-6 has shown beneficial effects by reducing inflammation and fibrosis in mice subjected to high salt intake after nephrectomy [56,57]. Interleukin-33 (IL-33) has been associated with pro-fibrotic signaling in rats with MI and in individuals diagnosed with HF. IL-4 has been identified as a contributor to heart fibrosis, as it stimulates pro-fibrotic activity in macrophages [58,59,60].

### 6.3. MicroRNA

The development of fibrosis in patients with diabetes or post-AMI may be attributed to pathophysiological processes, which may include the dysfunction of microRNA (miRNA, miR). These are tiny, single-stranded, non-coding RNAs that control gene expression after the transcription process. miRNAs have widespread expression in both the nucleus and cytoplasm. Nevertheless, miRNAs may still be detected in the bloodstream. They have a significant impact on several biological processes such as cell growth, specialization, programmed cell death, and metabolism by either inhibiting or promoting the expression of genes. Due to their stable expression in blood, miRNAs have the potential to serve as biomarkers for diagnosing, predicting outcomes, and developing new treatment approaches for cardiovascular disease [61,62,63].

In individuals with diabetes, there is an abnormal regulation of miRNA expression in the serum or plasma. Each miRNA has one or many targets that play a role in the development of diabetic fibrosis in the heart. Each gene might potentially be associated with one or many miRNAs, which can then inhibit its expression. Cardiac fibroblasts, endothelial cells, and cardiomyocytes have a role in the development of cardiac fibrosis. Every microRNA has a distinct target in certain cells, but many microRNAs have several targets in diverse cells. Mimicking of microRNAs inhibits the production of molecules of interest. Research indicates that cardiac fibrosis may include the activation of many crucial signaling pathways to enhance the production of pro-fibrotic molecules or hinder the production of anti-fibrotic molecules at the levels of gene expression and protein synthesis. miRNAs selectively target these molecules to exert an influence on the development of cardiac fibrosis in the diabetic heart or after a MI [64]. Several miRNAs have a function in reducing fibrosis in the diabetic heart affected by cardiac fibrosis. Those are represented by miR-15a/b, miR-18a-5p, miR-20a-5p, miR-26b-5p, miR-29, miR-133a, miR-141, miR-146a, miR-200b, miR-203, miR-222, and miR-551b-5p. Increased production of these miRNAs with anti-fibrotic properties inhibits the development of cardiac fibrosis in vivo and decreases the levels of molecules that promote fibrosis [65,66,67,68,69,70,71,72,73,74,75,76].

In addition, some miRNAs are increased in expression in the presence of high blood sugar levels. Typically, their specific objectives are genes or proteins that are suppressed in the heart of individuals with diabetes or in cells that are exposed to high levels of glucose. Myocardial fibrosis and heightened production of pro-fibrotic molecules are a consequence of overproduction of these miRNAs (miR-21–5p, miR-21–3p, miR-150–5p, miR-155, miR-221–3p, miR-223, miR-451). Antagomirs of these molecules, which are modified antisense oligonucleotides designed to target certain mature miRNA sequences, have been shown to provide cardiac protection and decrease cardiac fibrosis [77,78,79,80,81,82,83,84,85,86,87].

Following a MI, it has been proposed that miRNAs target many important fibrogenic pathways, including TGF-b/Smad, PTEN/Akt, angiotensin II/MAPK, or PIGF/VEGF-A. A full understanding of the function of miRNAs in fibrotic circumstances is hindered by the intricate nature of their activities in many cell types, as well as the extensive array of their molecular targets. Research findings indicate that certain miRNAs, such as miR-1, miR-19b, and miR-590-3p, could have a role in suppressing cardiac fibrosis by inhibiting the pathways triggered by fibrogenic growth factors or matricellular proteins [88,89,90]. The suppression of miR-34a and miR-1955 inhibits the pro-fibrogenic effects of TGF-β1 or those caused by angiotensin II in cardiac fibroblasts [91,92]. On the other hand, it has been proposed that miR-21, miR-92a, miR-195, miR-200a-3p, miR-125b, miR-34a, miR-146b-5p and miR-144-3p have fibrogenic effects [91,93,94,95,96,97,98,99,100,101].

The primary goal of miRNA-based therapeutics post-MI is to reinstate miRNA expression levels. According to the findings, administering a miR-21 mimic to mice with cardiac macrophages changed their condition from one that promotes inflammation to one that reduces inflammatory processes. This alteration led to an increase in angiogenesis, a decrease in apoptosis, and abnormal restructuring in the infarct-affected region [102]. Cardiomyocyte apoptosis is closely associated with the suppression of a particular gene known as protein phosphatase 1 regulatory subunit 10 or PNUTS. A microRNA known as miR-34a controls the expression of this gene. In mice, PNUTS improves function recovery following a heart attack [103]. Other therapeutic targets for miRNAs and exosomal miRNAs in MI are represented by miR-210, miR-15, exosomal miR-4943p, and miR-132 [104,105,106,107].

Future research should focus on identifying miRNAs as biomarkers and therapeutic targets that are common to various components of the metabolic illness group or ischemic disease.

## 7. Insulin Resistance and Hyperinsulinemia

Diabetic cardiomyopathy (DC) is a critical complication of DM marked by structural and functional impairments in the heart muscle, occurring without typical cardiovascular issues like coronary artery disease or valve disorders. It primarily develops due to prolonged high levels of hemoglobin A1c (HbA1c), a long duration of diabetes, and older age [108,109].

The etiology of DC is complex, involving multiple cellular and molecular factors that contribute to heart dysfunction. These include the overproduction of advanced glycation end-products (AGEs), activation of the hexosamine biosynthetic pathway, lipotoxicity, mitochondrial dysfunction, increased oxidative stress, and activation of the renin–angiotensin system and calcium homeostasis. These processes compound the effects of hyperglycemia, hyperinsulinemia, and insulin resistance, exacerbating the structural and functional decline of the heart in individuals with diabetes [110].

Prolonged high blood sugar levels in individuals with diabetes lead to increased insulin resistance and higher insulin levels, causing the death of heart muscle cells and the formation of scar tissue in the heart. This condition also impairs glucose processing, reduces glucose clearance, and increases gluconeogenesis. In diabetic cardiomyopathy, the diminished functionality of glucose transporters GLUT1 and GLUT4 in cardiac cells further disrupts glucose absorption and metabolism, which normally meets only 30% to 40% of the energy requirements for heart functions through glycolysis [111,112]. There is also an intense proliferation of cardiac fibroblasts, key players in initiating cardiac fibrosis, and an increase in ECM synthesis. AGEs, formed from prolonged glucose exposure, bind to receptors on heart cells, triggering collagen production and other ECM proteins through pathways like TGFβ, ultimately exacerbating cardiac dysfunction through fibrosis. Furthermore, the AGEs-RAGE signaling pathway activates inflammatory responses and promotes interstitial fibrosis, while lipid-related metabolic changes further impair cardiac function by promoting cardiomyocyte death and reducing myocardial energy efficiency [113,114,115].

Oxidative stress plays a pivotal role in the development and progression of DC, primarily through the increased production of ROS and a reduction in antioxidant defenses such as glutathione peroxidase and superoxide dismutase, leading to heart fibrosis and mitochondrial damage. These oxidative changes impair mitochondrial function and calcium handling within cells, further reducing cardiac efficiency. Additionally, persistent ROS activity activates the TGFβ1/Smad3 signaling pathway, which enhances fibrotic changes in the heart by upregulating fibrotic markers and promoting the growth of cardiac fibroblasts, contributing significantly to the structural and functional decline observed in DC [116,117].

In individuals with diabetes, the chronic activation of the renin–angiotensin–aldosterone (RAS) pathway leads to the increased levels of angiotensin II (AT-II), which promotes left ventricular hypertrophy, vasoconstriction, cardiac dysfunction through fibrosis, oxidative stress, and cardiomyocyte death. This activation, further enhanced by elevated glucose, AGEs, and ROS, results in increased collagen production and decreased breakdown, contributing to the progression of cardiac fibrosis in DC [110,118].

In DM, identified as an inflammatory condition, the activation of pro-inflammatory genes and proteins, particularly through nuclear factor-KB (NF-kB), plays a crucial role in cardiac damage. NF-kB promotes the expression of pro-inflammatory cytokines and the NLRP3 inflammasome in the heart, leading to a self-perpetuating cycle of inflammation exacerbated by elevated ROS and the pro-inflammatory activation of monocytes/macrophages [119,120,121].

Inflammation, oxidative stress, activated RAS, compromised mitochondrial function, and disrupted calcium handling drive the structural and functional deterioration observed in DC.

## 8. Novel Drugs and Their Anti-Fibrotic Role

### 8.1. SGLT2 Inhibitors

SGLT2i works by enhancing glucose excretion in urine, primarily through inhibiting SGLT2, thereby reducing the renal threshold value of glucose and decreasing the reabsorption of filtered glucose. However, the benefits observed in HF associated with SGLT2i are unlikely to be solely attributed to glucose lowering. Rather, the advantages for CV and renal health have been linked to various mechanisms including hemodynamic effects, anti-inflammatory properties, anti-fibrotic effects, antioxidant actions, reno-protective effects, attenuation of glucotoxicity, reduction in uric acid levels, modification of the neurohumoral system, and cardiac metabolism [6,122,123,124]. While the CV benefits of SGLT2i are evident, the precise mechanisms underlying these effects are not fully understood.

SGLT2i plays a significant role in managing blood glucose levels by specifically targeting the SGLT2 transporter in the proximal renal tubules, leading to decreased glucose reabsorption and increased glucose excretion in the urine. This mechanism effectively reduces blood glucose concentrations independent of insulin pathways, thereby not triggering insulin release or causing hypoglycemia. Consequently, this reduction in circulating glucose helps to improve insulin sensitivity in muscle tissues and enhances β-cell function, contributing to the management of insulin resistance in patients with diabetes [125,126,127,128,129].

ACS is characterized by coronary artery obstruction, leading to myocardial ischemic damage, which can have life-threatening consequences. Immediate treatment is provided to restore oxygenation to the affected tissue, and patients are often prescribed secondary preventive medications to reduce the risk of future ACS events. Despite consistent prescription practices over the years, there is potential to include SGLT2i in this regimen due to their demonstrated CV benefits. Clinical research on the impact of SGLT2i on CV health in patients after MI has yielded conflicting results, with most trials focusing on MACEs rather than the underlying cellular processes including inflammation, cardiac metabolism, or cardiac fibrosis.

Inflammation plays a central role in myocardial fibrosis development, involving various inflammatory factors. However, it remains uncertain whether SGLT2i influences the inflammatory response and whether they can impede myocardial fibrosis progression by targeting inflammation. Wang et al. found that dapagliflozin effectively reduced levels of TNF-α, IL-1β, IL-6, and hs-CRP in patients with diabetes and HF, leading to a gradual decline in inflammation and alleviation of myocardial fibrosis [130]. Dapagliflozin also reduced cardiac fibrosis in T2DM rats by inhibiting endothelial-to-mesenchymal transition (EndMT) and fibroblast activation via AMPKα activation, which suppressed the TGF-β/Smad signaling pathway [131]. Dapagliflozin decreased oxidative stress in the heart and prevented the production of inflammatory substances, thereby inhibiting myocardial enlargement and scar tissue formation [132,133,134]. Additionally, via raising AMPK phosphorylation, it inhibits high glucose-induced endothelial cell dysfunction [135]. A recent clinical trial has shown the efficacy of dapagliflozin in improving glycemic fluctuations and reducing oxidative stress in individuals with T2DM [136]. Recent investigations have shown that dapagliflozin provides protection against myocardial ischemia/reperfusion injury (IRI) via restricting the activation of the NLRP3 inflammasome [137].

In mouse models, empagliflozin was found to reduce myocardial infarct size and preserve myocardial function after ischemia/reperfusion injury by regulating inflammatory responses and redox signaling in the ischemic myocardium [138,139]. Empagliflozin also reduced cardiomyocyte hypertrophy, interstitial fibrosis, and oxidative stress in non-diabetic rat models of HF [140,141]. However, long-term empagliflozin administration did not significantly improve LV function, shape, adiposity, or diffuse fibrosis in individuals with T2DM [142]. Similarly, no significant difference in mortality was observed between patients with diabetes receiving low-dose empagliflozin after percutaneous coronary intervention (PCI) for ACS and the placebo group [143]. Nonetheless, empagliflozin treatment improved heart function in mice with diabetes after infarction, particularly diastolic function, suggesting potential benefits in diabetic cardiomyopathy [144]. Nrf2 has a crucial role as a transcription factor in regulating the expression of genes that are involved in antioxidant and cytoprotective functions. Empagliflozin enhances Nrf2 expression and facilitates its nuclear translocation, therefore mitigating heart dysfunction in db/db mice with diabetes via the amelioration of oxidative stress and mitochondrial dysfunction [145,146].

Furthermore, SGLT2i such as dapagliflozin, canagliflozin, and empagliflozin have been shown to have anti-inflammatory effects by reducing the production of the NLRP3 inflammasome, IL-1β, IL-6, TNF-α, and LV ECM remodeling in diabetes animal models [137,147,148,149,150,151]. In the same line, Hodrea, J. et al. demonstrated that dapagliflozin effectively decreased the levels of IL-1β, IL-6, and TNF-α in the LV of rats with type 1 diabetes mellitus (T1DM), indicating its potential as an anti-inflammatory agent [149]. Empagliflozin attenuates TGF-β1-induced fibroblast activation and decreases pro-fibrotic markers such as type I collagen (COL-1), connective tissue growth factor (CTGF), and MMP-2 [152]. Additionally, it has been shown to alleviate cardiac fibrosis by downregulating the TGF-β/Smad pathway, triggering Nrf2/ARE signaling, and lowering the concentrations of Smad1, Smad2, and Smad3 in the heart tissue of mice with diabetes [146].

According to recent research, SGLT2i may have a role in LV remodeling in individuals with diabetes. Following this therapy, individuals with T2DM with or without CAD showed a decrease in LV mass measured by cardiac MRI, as stated by two randomized trials [153,154]. Recent results indicate that the treatment of SGLT2i to individuals with diabetes resulted in a decrease in blood hs-CRP levels and an improvement in endothelial function. In contrast, the EMBLEM study found that a 24-week therapy with empagliflozin did not lead to any improvement in endothelial dysfunction in patients with preexisting cardiovascular disease and T2DM [155,156,157,158]. Collectively, these findings suggest that SGLT2i may have potential therapeutic effects beyond glucose lowering, particularly in mitigating inflammation and fibrosis in CV diseases.

### 8.2. Glucagon-like Peptide-1 Receptor Agonists

Glucagon-like peptide-1 (GLP-1) is a hormone naturally produced and released by endocrine L-cells in the small intestine upon food ingestion. It binds to specific GLP-1 receptors in the pancreas, stimulating insulin release in a glucose dependent manner. Despite its widespread presence in various organs, natural GLP-1 has a short half-life due to rapid breakdown by dipeptidyl peptidase-4 (DPP-4) [159,160,161].

Incretin hormones significantly influence insulin production post-meal, accounting for 50–70% of total insulin release, but their effect is reduced in patients with T2DM due to various factors. These hormones, particularly GLP-1, enhance insulin secretion, delay stomach emptying, and suppress glucagon release, helping regulate postprandial blood glucose levels. To augment the actions of GLP-1, two therapeutic strategies are employed and are as follows: GLP-1RAs, which resist the degradation by DPP-4 enzyme and thus prolong receptor activation, and DPP-4 inhibitors, which extend the life of endogenous incretin hormones. GLP-1RAs improve glucose-dependent insulin release, reduce glucagon secretion, enhance insulin sensitivity, and have additional benefits on appetite suppression, cardiovascular protection, renal sodium excretion, and metabolic functions in adipose and muscle tissues. The efficacy of GLP-1RAs varies with their duration of action; long-acting forms are particularly effective in controlling both postprandial and fasting glucose levels, demonstrating significant improvements in glycemic control in patients with T2DM [162,163,164,165].

Studies have shown that GLP-1RAs can modulate ECM homeostasis by inhibiting the mitogen-activated protein kinase (MAPK) pathway and BATF/JUN heterodimer formation. They also downregulate the pro-fibrotic factor TGF-β1, suppressing SMAD3 and extracellular signal-regulated kinase 1/2 (ERK1/2) signaling while enhancing WNT/β-catenin signaling and other pathways, thus reducing extracellular matrix protein deposition and delaying fibrosis [166,167,168].

GLP-1RAs ameliorate diabetes-related cardiac fibrosis by stimulating PPARδ expression. Additionally, GLP-1 attenuates high glucose-induced ROS production in cardiomyocytes through an AMPK/mTORC1/p70S6K-dependent mechanism [169,170]. In vitro, semaglutide suppressed cardiomyocyte apoptosis produced by ischemia/reperfusion (I/R) via activating the PKG/PKCε/ERK1/2 pathway [171]. It also might potentially be a beneficial treatment target for preventing diabetic cardiomyopathy by modifying integrin-linked kinase (ILK)-associated TGF-β/SMAD signaling pathways [172].

Research has shown that GLP-1 analogs, such as liraglutide, may prevent myocardial damage by activating the Sirt1/AMPK signaling pathways to suppress cellular pyroptosis in diabetic cardiac tissues and by lowering the expression of fibrotic markers [173,174]. Moreover, treatment with liraglutide limits infarct size, expansion, and cardiac rupture in the normal and diabetic heart by activating prosurvival kinases and cytoprotective genes in the heart in a preclinical murine model of experimental ischemia following coronary artery obstruction. In mouse cardiomyocytes cultivated in vitro, it also raises cAMP and lowers apoptosis [175].

GLP-1 analog exendin-4 has been found to suppress fibrosis and PPARα-mediated lipid accumulation via the protein kinase A/Rho-associated protein kinase (PKA/ROCK) pathway [176]. Recent studies in vitro have shown that exendin-4 effectively reduces the formation of intracellular and mitochondrial ROS, prevents apoptosis, and improves mitochondrial membrane potential impairment. Additionally, it activates genes associated with mitochondrial functions and dynamics via the cAMP/Epac/PI3K/Akt pathway. By encouraging the nuclear translocation of TFEB, it restores autophagic flux. It also protects endothelial function by upregulating the expression of HDL scavenger receptor class BI via the AMPK/FoxO1 pathway and eNOS activation. Lastly, it decreases the size of infarcts and maintains cardiac function by having anti-apoptotic and antioxidative properties [177,178,179]. Research have demonstrated the cardioprotective benefits of exendin-4 in rats with diabetes. This is due to its capacity to enhance mitochondrial activities by targeting inflammatory and autophagy processes and suppressing cardiomyocyte pyroptosis via the AMPK-TXNIP pathway [180,181]. In mice models, after MI, the positive impacts of exendin-4 on cardiac remodeling may be achieved via activating the eNOS/cGMP/PKG pathway, β-arrestin-2, and protein phosphatase 2A and suppressing β-catenin, collagen I, collagen III, transforming growth factor-β1, and PI3K/AKT signaling pathway [181,182,183,184].

Modified mesenchymal stem cells engineered to secrete a fusion protein have demonstrated positive effects on cardiac recovery post-infarction by reducing TGF-β expression and influencing extracellular matrix protein deposition. GLP-1RAs inhibit cardiac remodeling in rats after AMI by modulating various signaling pathways [182,185]. In murine models, liraglutide reduces cardiac fibrosis and enhances capillary density, potentially improving heart function [186,187].

Clinical studies have also shown the anti-fibrotic effects of GLP-1RAs. Reduced GLP-1 levels in plasma have been associated with atheroma instability and cardiac fibrosis in non-diabetic patients with ACS [188]. Initiating GLP-1RAs therapy shortly after a first AMI in patients with diabetes has been linked to a reduced risk of significant CV events [189]. Long-term treatment with GLP-1RAs and/or SGLT-2i positively impacts the clinical prognosis of patients with diabetes that are hospitalized with AMI [190,191].

However, conflicting results exist in clinical trials regarding the impact of GLP-1RAs on myocardial remodeling. While some studies show potential benefits, others reveal no significant effects on cardiac function or fibrosis parameters [192,193]. Nonetheless, retrospective studies suggest a reduced risk of CV events and mortality in patients with diabetes treated with GLP-1RAs [194].

In summary, the potential anti-fibrotic effects of GLP-1RAs offer promising avenues for clinical treatment by inhibiting epithelial–endothelial transformation, suppressing inflammatory responses, and reducing oxidative stress damage while controlling blood glucose levels.

### 8.3. Dipeptidyl Peptidase-4 Inhibitors

DPP4i are small, orally administered compounds that specifically interact with the catalytic site of DPP4 without affecting its other known activities, such as its immunomodulatory effects. DPP-4 is a serine protease primarily responsible for deactivating the GLP-1 and GIP generated in the intestines. The efficacy of DPP4i as anti-diabetic agents is closely tied to their ability to inhibit DPP4 enzyme activity, thus leading to a prolongation of the lifespan of incretin hormones and an improvement in insulin production, which is reliant on glucose levels. By prolonging the half-life of GLP-1, increasing insulin levels, and lowering blood glucose levels, DPP4i offer therapeutic benefits [195,196]. Contrary to the anti-diabetic medications stated above, DPP-4 inhibitors do not impact insulin sensitivity or secretion [197,198,199,200].

A meta-analysis has shown that DPP-4 inhibitors may effectively reduce low-grade inflammation in individuals with T2DM by decreasing levels of C-reactive protein (CRP), TNF-α, IL-6, and IL-1β [201]. Recent studies on diabetic animal models, such as db/db mice, have demonstrated the cardioprotective effects of DPP4 inhibitors like evogliptin. These inhibitors mitigate the primary drivers of diabetic cardiomyopathy, including cardiac hypertrophy, fibrosis, and systolic and diastolic dysfunction. Evogliptin has been shown to reduce mitochondrial dysfunction and lipotoxicity by reducing myocardial fat formation or modulating transcription factors [202]. Additionally, gemigliptin was also shown to reduce cardiomyocyte cross-sectional areas and interstitial and perivascular fibrosis [203]. In rats with HF, monotherapy with DPP4i like valsartan and vildagliptin has been found to decrease LV hypertrophy, alleviate cardiac interstitial fibrosis, and improve systolic and diastolic performance [204].

Studies by Wójcicka et al. investigated whether incretin drugs like exenatide and sitagliptin exert heart-protective and anti-fibrotic effects through their influence on asymmetric dimethylarginine (ADMA) metabolism. Both drugs reduced cardiac fibrosis and immune response around blood vessels. In metabolic syndrome, sitagliptin and exenatide positively impacted cardiac fibrotic remodeling and circulating levels of endogenous NOS inhibitors, such as ADMA, which inhibit NO production, essential for maintaining healthy blood vessels [205]. Additionally, DPP4i have been found to have antifibrotic effects by blocking the interaction between membrane-bound DPP-4, integrin, and the caveolin complex [206].

Linagliptin improved systolic and diastolic blood pressure and cardiac function while reducing cardiac fibrosis in animal models. It also ameliorated structural disruptions in the heart induced by diabetic conditions [207]. In db/db mice with MI, it targeted the NLRP3/ASC inflammasome, increasing EF and lowering IL-1β and IL-6 levels [208].

Sitagliptin treatment effectively hindered cardiac fibrosis development and alleviated cardiac hypertrophy [209]. It also mitigates heart dysfunction and unfavorable remodeling after AMI in diabetic animal models [210]. Additionally, it reduced the severity of diabetic cardiomyopathy by suppressing the JAK/STAT signaling pathway and decreasing TGF-β1 protein levels, therefore avoiding myocardial remodeling [211,212].

Alogliptin reduced interstitial fibrosis and decreased mitochondrial ROS generation, preventing the mitochondrial depolarization of membranes and swelling in rabbits with diabetes [213,214]. Interestingly, adding saxagliptin to dapagliflozinin may have reduced the degree of activation of the NLRP3/ASC inflammasome. Higher myocardial lipid deposits, oxidative stress, and apoptosis were all reduced by saxagliptin [150,176].

Despite several CV outcome trials evaluating the efficacy and safety of new glucose-lowering drugs for CV outcomes, there is limited research on their correlation with the reduction in cardiac fibrosis risk in patients with diabetes and ACS.

### 8.4. Neprilysin Inhibitor

Neutral endopeptidase, also known as neprilysin (NEP), breaks down natriuretic peptides (NPs) by hydrolysis. Neprilysin inhibitors (NEPis) have been discovered to elevate the concentration of defensive natriuretic peptides (NPs), hence enhancing their ability to defend against diabetic consequences. Nevertheless, NEPi alone proved inadequate in addressing cardiovascular disorders as mandated by conventional pharmacology, prompting the development of dual-acting inhibitors that target both neprilysin and the pressor arm (Ang II/ACE/AT1R) of the RAS. Recent human trials have shown that the dual angiotensin receptor–neprilysin inhibitor (ARNi) has been effective in enhancing glycemic control and insulin sensitivity in persons with T2DM and/or obesity. Additionally, preclinical research has also shown that inhibiting neprilysin, either on its own or in conjunction with renin–angiotensin system blockers, has positive effects on glucose regulation [215,216,217].

Studies have shown that ARNIs enhance glucose homeostasis, glucose management, and insulin sensitivity in individuals with T2DM [215]. Evidence from human research substantiates the efficacy of neprilysin inhibitors for both preventing and treating type 2 diabetes. The PARADIGM-HF study shown that administering the ARNi over a duration of 3 years led to a more significant decrease in HbA1c levels and a reduced number of patients needing to start oral glucose-lowering medicines or insulin therapy, in comparison to using just an ACE inhibitor. In an additional investigation, switching the therapy from either an ACE inhibitor or ARB to ARNi for a duration of 3 months led to a drop in serum neprilysin activity. This decrease was shown to be linked to lower levels of fructosamine, a marker used to measure protein glycation. The results of this study, along with a previous publication, indicated that ARNi may have positive effects on glycemic management by partially raising the levels of GLP-1 in the bloodstream [218,219].

In streptozotocin (STZ)-induced mice with diabetes and HF and a decreased EF, LCZ696, the initial member of ARNi, has been shown to enhance cardiac functions and mitigate fibrosis by inhibiting TGF-ß [220]. According to research by Qing et al., mice with diabetes treated with LCZ696 had better cardiac function and ventricular remodeling than mice with diabetes that were left untreated. Additionally, LCZ696 reduced oxidative stress in vitro and in vivo. Finally, via lowering oxidative stress, apoptosis, and cardiac inflammation, LCZ696 ameliorated diabetic cardiomyopathy [221].

Researchers have created and tested omapatrilat, the most researched vasopeptidase inhibitor, against heart diseases associated with diabetes. Compared to ACEi, this medication had greater cardioprotective potential in spontaneously diabetic rats because it controlled systolic blood pressure, improved diastolic function, and decreased heart hypertrophy [222]. By restoring RAS and NPS activities, raising cGMP, and suppressing inflammatory, profibrotic, and apoptotic signaling, a recent study demonstrated the cardioprotective efficacy of NEPi and ACE2 activator combination treatment, which was successful in preventing DC [223].

In conclusion, not many studies have been conducted to determine how neprylisin inhibitors affect DC. Additional research is required to investigate the possible processes that underlie its effects, since this might serve as the foundation for a novel treatment approach for it.

### 8.5. Sarcoplasmic/Endoplasmic Reticulum Calcium ATPase

The pathological and molecular processes that underlie diabetes include irregularities in the control of calcium homeostasis within cardiomyocytes, which subsequently modifies the ventricular excitation–contraction coupling. A dysregulation of Ca2+ cycling in the diabetic heart involves a decrease in sarcoplasmic/endoplasmic reticulum calcium ATPase (SERCA) activity, which may also be accompanied by a drop in SERCA protein expression. Multiple preclinical and clinical experiments have shown that maintaining proper management of blood sugar levels is ineffective in slowing the course of diabetic cardiomyopathy, particularly in the intermediate and advanced phases. Thus, individuals with diabetic cardiomyopathy may continue to experience hyperglycemic stress despite the careful management of blood glucose levels. The etiology of DC is mostly investigated utilizing tiny molecular probes that have strong effectiveness against it. As a result, SERCA could be a target molecule for pharmacological intervention targeted at improving the mechanical performance and energy efficiency of the diabetic heart, which is characterized by a malfunctioning SR Ca2+ loading [224].

Recent research has shown that SJ-12, a new curcumin derivative, has therapeutic efficacy in STZ-induced diabetic cardiomiopathy. Furthermore, SJ-12 administration may reduce the fibrotic response. SJ-12 functions as an anti-fibrosis agent by specifically targeting the OGT-regulated O-GlcNAc-Sp1/SERCA2a/calcium signaling pathway [225].

A significant buildup of collagen and ECM proteins, linked to higher death rates, defines cardiac fibrosis. Recent findings suggest that the deregulation of SERCA2a and inappropriate handling of Ca2+ cause cardiac fibrosis. An imbalance in Ca2+ levels, on the other hand, can cause problems with energy metabolism, which in turn speeds up the development of cardiac fibrosis by making more matrix proteins. Conventional autophagy is responsible for breaking down and reusing cellular components, which helps to sustain cellular energy production. Excessive or inadequate autophagy may have a detrimental effect on the viability of cardiomyocytes, leading to a deterioration in both the structure and function of the heart. Latest research has shown that inhibiting the expression of SERCA might serve as a trigger for endoplasmic reticulum (ER) stress, which in turn leads to excessive autophagy and eventually results in cardiac fibrosis [226,227,228].

Most of the studies have mostly focused on the function of SERCA as the calcium pump responsible for transporting calcium from the cytosol to the SR lumen, working against the concentration gradient. However, there is little information about its involvement in cardiac fibrosis in patients with diabetes.

In Table 1, the main studies evaluating the antifibrotic effects of hypoglycemic medication are depicted.

## 9. Limitations and Future Directions

While this research offers valuable insights, it is important to acknowledge certain limitations that may affect the interpretation and generalization of findings. Firstly, many studies assessing the effectiveness of anti-diabetic medications in reducing fibrosis risk post-MI have relatively short follow-up periods, limiting our understanding of their long-term effects. Secondly, variability in patient characteristics, such as age, comorbidities, and medication history, across different studies may affect the generalizability of findings. Finally, while some studies demonstrate the efficacy of anti-diabetic medications in reducing fibrosis, the exact mechanisms underlying these effects remain incompletely understood.

Thus, we recognize the need for future research to focus on addressing these limitations, exploring new directions, and contributing to advancing our knowledge in the field. Further research is needed to elucidate the precise mechanisms through which anti-diabetic medications exert their anti-fibrotic effects post-MI, potentially uncovering novel therapeutic targets. Longitudinal studies with extended follow-up periods are necessary to assess the sustained efficacy and safety of anti-diabetic medications in reducing fibrosis and improving CV outcomes. Investigating the potential synergistic effects of combining anti-diabetic medications with other therapeutic agents targeting different pathways involved in fibrosis could lead to enhanced treatment efficacy. In conclusion, future research should focus on elucidating the underlying mechanisms of action of these medications, conducting longitudinal studies with extended follow-up periods, and exploring personalized medicine approaches and combination therapies.

## 10. Conclusions

In conclusion, myocardial fibrosis is a critical contributor to adverse cardiac remodeling following AMI, particularly in patients with DM. Emerging evidence suggests that anti-diabetic medications, such as SGLT2i, GLP-1RAs, and DPP4i, may hold promise in attenuating fibrosis and improving CV outcomes in this patient population. Overall, the identification of effective anti-fibrotic strategies in patients with diabetes post-MI holds significant clinical implications for improving CV outcomes and reducing the burden of heart failure. Continued research efforts in this area are essential for advancing our understanding and refining treatment strategies to optimize patient care.

## Figures and Tables

**Table 1:** The main antifibrotic effect of novel medication.

Novel Medication	Antifibrotic Effect
**SGLT2i**	Reduces the levels of TNF- α, IL-1β, IL-6, and hs-CRP in patients with HF and diabetes [130]. Reduces cardiac fibrosis in rats with T2DM by inhibiting EndMT and fibroblast activation [131]. Decreases oxidative stress and prevents the production of TNF-α, IL-1β, IL-6, and MCP-1 [132,133,134]. Increases the phosphorylation and expression of STAT3 while reducing the expression of myocardial IL-6 and iNOS [138]. Increases the activation of M2 macrophages, leading to a reduction in cardiac fibrosis at a molecular level [139]. Decreases the production of the NLRP3 inflammasome, IL-1β, IL-6, and TNF-α [137,147,148]. Attenuates TFG-β1-induced fibroblast activation and decreases COL-1, connective tissue growth factor, and MMP-2 [152]. Alleviates cardiac fibrosis by downregulating the TGF-β/Smad pathway, triggering Nrf2/ARE signaling, and lowering the concentrations of Smad1, Smad2, and Smad3 in the heart tissue of mice with diabetes [146]. Effectively decreases the levels of IL-1β, IL-6, and TNF-α in the LV of rats with T1DM [149]. Enhances Nrf2 expression and facilitates its nuclear translocation, ameliorating oxidative stress and mitochondrial dysfunction [145].
**GLP-1RAs**	By stimulating the expression of PPARδ, GLP-1RAs ameliorate diabetes-related cardiac fibrosis in a diabetic cardiomyopathy model [169]. Decrease high glucose-induced ROS production in cardiomyocytes via a mechanism that is reliant on AMPK/mTORC1/p70S6K [170]. Lower the expressions of P4hα-1, COL-1, COL-3, MMP-1, and MMP-9 [173]. Suppress fibrosis and PPARα-mediated lipid accumulation and toxicity controlled by the PKA/ROCK pathway [176]. Reduce cardiac remodeling in rats after AMI by inhibiting the activation of β-arrestin-2, PP2A, and GSK3β [182,185]. Suppress the production of CTGF in both the infarcted and non-infarcted regions of mice after a heart attack by stimulating cAMP and decreasing the migration of collagen fibers from the infarcted region to the non-infarcted region [186,187]. Suppress cardiomyocyte apoptosis produced by ischemia/reperfusion (I/R) via activating the PKG/PKCε/ERK1/2 pathway [171]. Prevent myocardial damage by activating the Sirt1/AMPK signaling pathways to suppress cellular pyroptosis in diabetic cardiac tissues [173,174]. Effectively reduce the formation of intracellular and mitochondrial ROS, prevent apoptosis, and improve mitochondrial membrane potential impairment [177,178,179]. Activate genes associated with mitochondrial functions and dynamics via the cAMP/Epac/PI3K/Akt pathway [177,178,179]. Enhance mitochondrial activities by targeting inflammatory and autophagy processes and suppressing cardiomyocyte pyroptosis via the AMPK-TXNIP pathway [180,181].
**DPP4i**	Decreases LV hypertrophy, relieves cardiac interstitial fibrosis, and enhances systolic and diastolic performance [204]. Decreases the amount of fibrosis around blood vessels and has positive impact on cardiac fibrotic remodeling and the circulating level of endogenous NOS inhibitors [205]. Exhibits antifibrotic effects by blocking the interaction between membrane-bound DPP-4, integrin, and the caveolin complex [206]. Linagliptin exhibits improvements by reducing disruptions in myocardial fiber, mitochondria, and sarcomere, along with the necroptosis [207]. Sitagliptin treatment effectively hinders the development of cardiac fibrosis and alleviates cardiac hypertrophy [209]. Reduces the severity of diabetic cardiomyopathy by suppressing the JAK/STAT signaling pathway and decreasing TGF-β1 protein levels, therefore avoiding myocardial remodeling [211,212].
**ARNi**	Enhances cardiac functions and mitigates fibrosis by inhibiting TGF-ß [220]

HF = heart failure; LV = left ventricle; TNF-α = tumor necrosis factor-α; IL-1β = interleukin-1β; IL-6 = interleukin-6; hs-CRP = high sensitivity c-reactive protein; COL-1 = type 1 collagen; COL-3 = type 3 collagen; MMP-1 = matrix metalloproteinase 1; MMP-2 = matrix metalloproteinase 2; MMP-9 = matrix metalloproteinase 9; PPARα = peroxisome proliferator-activated receptor alpha; PKA/ROCK = protein kinase A/Rho-associated protein kinase; PP2A = protein phosphatase 2; DPP4 = dipeptidyl peptidase-4; DPP4i = dipeptidyl peptidase-4 inhibitor; SGLT2i = sodium-glucose cotransporter 2 inhibitors; GLP-1RAs = glucagon-like peptide-1 receptor agonists; CTGF = connective tissue growth factor; cAMP = cyclic adenosine monophosphate; GSK3β = glycogen synthase kinase 3 beta; STAT3 = signal transducer and activator of transcription 3; iNOS = inducible form of nitric oxide synthase; P4hα-1 = prolyl 4-hydroxylase subunit alpha 1; EndMT = endothelial-to-mesenchymal transition; ARNi = angiotensin receptor–neprilysin inhibitor; and T1DM = type 1 diabetes mellitus.
